# *In vitro* human colonic microbiota utilises D-β-hydroxybutyrate to increase butyrogenesis

**DOI:** 10.1038/s41598-020-65561-5

**Published:** 2020-05-22

**Authors:** Kengo Sasaki, Daisuke Sasaki, Asuka Hannya, Jun Tsubota, Akihiko Kondo

**Affiliations:** 1 Graduate School of Science, Technology and Innovation, Kobe University, 1-1 Rokkodai-cho, Nada-ku, Kobe, Hyogo 657-8501 Japan; 2 Energy Technology Laboratories, OSAKA GAS CO., LTD., 6-19-9 Torishima, Konohana-ku, Osaka 554-0051 Japan; 3 RIKEN Center for Sustainable Resource Science, 1-7-22 Suehiro-cho, Tsurumi-ku, Yokohama, Kanagawa 230-0045 Japan

**Keywords:** Biotechnology, Signs and symptoms

## Abstract

The ketone body D-β-hydroxybutyrate (DBHB) has gained attention owing to its cellular signalling function; however, its effect on the human colonic microbiota remains unclear. Here, DBHB dynamics in the human colon were investigated using an *in vitro* colonic microbiota model, which maintained most of the operational taxonomic units detected in the original faeces. Over 54% of 0.41% (w/v) DBHB was metabolised by microbiota models originating from seven faecal samples after 30 h of fermentation (regarded as DBHB utilisers); however, <19% of DBHB was metabolised by microbiota models from five faecal samples (regarded as non-utilisers of DBHB). In utilisers, DBHB administration increased the relative abundance of the genus *Coprococcus*, correlated with increased butyrogenesis. Increased butyrogenesis was not observed in DBHB non-utilisers. Based on PICRUSt analysis, the relative abundance of β-hydroxybutyrate dehydrogenase was maintained in microbiota models from DBHB utilisers following DBHB administration; however, it decreased in microbiota models from non-utilisers. After 21 h of fermentation, the intracellular glutamate concentration, which is indicative of growth, showed a positive correlation with DBHB utilisation (R^2^ = 0.70). Human colonic microbiotas with high growth activity demonstrate efficient utilisation of DBHB for increased butyrate production, which affords health benefits.

## Introduction

Ketogenesis and ketolysis are central metabolic processes activated during the response to nutrient deprivation, which induces a shift from carbohydrate to fat utilisation^1^. In mammals, ketone bodies [D-β-hydroxybutyrate (DBHB), acetoacetate and acetone] are predominantly produced in the liver from acetyl-coenzyme A derived from fatty acid oxidation^2^. Ketone body oxidation becomes a significant contributor to overall mammalian energy metabolism within extrahepatic tissues, such as the brain, heart or skeletal muscle, in physiological states such as fasting and starvation^2^. Ketogenic diets, which are high in fat and low in carbohydrates, have been used to reduce the likelihood of epileptic seizures, characterised by recurrent involuntary brain activity, particularly in drug-resistant patients^3^. Ketogenic diets have also gained interest as potential treatments for other diet-sensitive neurological disorders such as malignant glioma and Alzheimer’s disease in adults^4^. However, ketogenic diets involving a high amount of fat are limited by patient compliance^5^. Consequently, exogenous supplementation of ketone bodies^6^, for example, using D,L-β-hydroxybutyrate (BHB) salt^7,8^, may be a viable alternative. Seizure onset time has been reported to be prolonged in a rat seizure model following BHB pretreatment^9^.

The interactions between the gut microbiota and the host immune system have an impact on health or disease risk^10^. Gut microbiota patterns in infants with refractory epilepsy significantly differed from those of healthy infants, demonstrating an accumulation of *Proteobacteria* species comprising a variety of notorious pathogens^11^. Previous studies have investigated how a ketogenic diet modifies the gut microbiota^12,13^. Consumption of ketogenic diets reduced the total gut microbial counts in a murine model of autism spectrum disorder^12^, and reduced the microbial compositional richness and diversity in children with refractory epilepsy^14^. Notably, the ketogenic diet enriched *Akkermansia muciniphila*^15^ and *Parabacteroides* in the mouse gut microbiota, which could reduce gamma-glutamylation activity, elevate hippocampal gamma-aminobutyric acid/glutamate levels and protect against seizures *in vivo*^13^. In addition, dietary poly-BHB has been reported to change the composition of the human intestinal microbiota^16^. However, the association between DBHB administration and the human colonic microbiota remains unclear.

The aim of the present study was to investigate the effect of DBHB administration on the human colonic microbiota. This was conducted using an anaerobic culturing system involving an *in vitro* human colonic microbiota model [hereafter referred to as the Kobe University Human Intestinal Microbiota Model (KUHIMM)], which is capable of closely reproducing the composition and diversity of the microbiota in the original faecal inoculum^17^.

## Results

### D-β-hydroxybutyrate utilisers and non-utilisers

**Figure 1:**
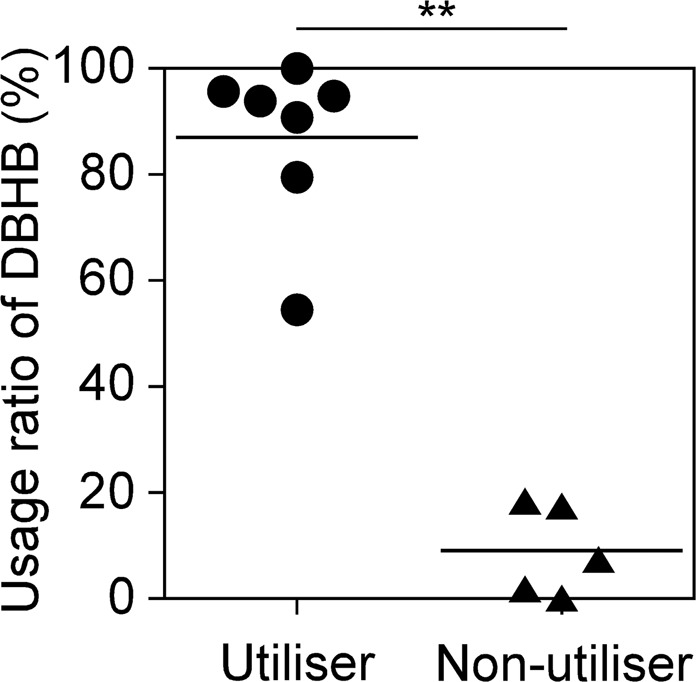
Usage ratio (%) of D-β-hydroxybutyrate (DBHB) {= 100 × [(Initial concentration) - (Concentration at 30 h)]/(Initial concentration)} in the *in vitro* human colonic microbiota model (KUHIMM). Each dot and triangle represent the usage ratio of one KUHIMM after 30 h of fermentation with the original faecal inoculum. **p < 0.01; Mann-Whitney U test.

DBHB availability was assessed using 12 *in vitro* human colonic microbiota models (KUHIMMs), with or without the addition of 0.5% (w/v) sodium DBHB; each KUHIMM was created from a faecal inoculum from 1 of 12 healthy individuals. At 30 h post fermentation initiation, the DBHB usage ratio was calculated for each KUHIMM by comparing the DBHB in the fermentation broth at 0 and 30 h (Fig. 1). Interestingly, the 12 KUHIMMs were divided into seven utilisers and five non-utilisers of DBHB (Mann-Whitney U test; *p* = 0.0058). In the seven utilisers, 54.5 to 100% of DBHB was metabolised. However, only 0 to 18.5% of DBHB was metabolised by the five non-utilisers.

### D-β-hydroxybutyrate increases the genus *Coprococcus* in utilisers

**Figure 2:**
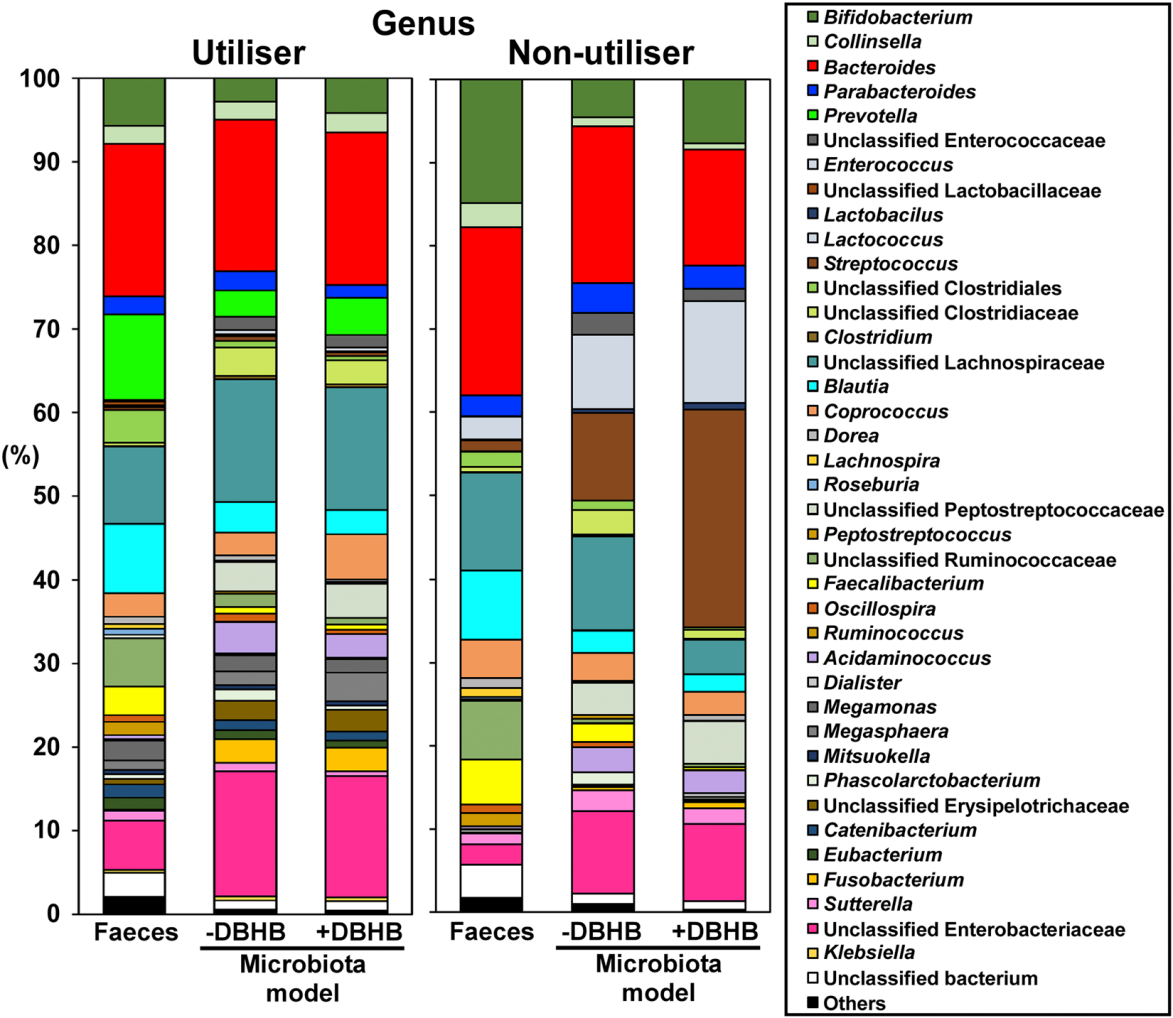
Genus-level classification of bacteria in the original faecal samples (Faeces), the corresponding *in vitro* human colonic microbiota model, KUHIMM (-DBHB) and the corresponding KUHIMM with D-β-hydroxybutyrate (+DBHB) after 30 h of fermentation with the original faecal inoculum. The averages of seven healthy human subjects, classified as DBHB utilisers, and three healthy human subjects, classified as DBHB non-utillisers, are shown.

We next investigated the effect of DBHB on human colonic microbiota using KUHIMMs with and without 0.5% (w/v) sodium DBHB. The V3-V4 region of the bacterial 16S rRNA gene was sequenced using the MiSeq system. For the seven DBHB utilisers, a total of 3,293,718 sequences were obtained from the seven faecal inoculums, the seven corresponding KUHIMMs and the seven corresponding KUHIMMs with DBHB administration after 30 h of fermentation (Table 1). Operational taxonomic unit (OTU) analysis yielded similar results among the faecal samples, corresponding KUHIMMs and corresponding KUHIMMs with DBHB administration (Mann-Whitney U test; *p* > 0.05). The alpha diversity estimates tested included Chao1 for richness and Shannon’s and Simpson’s evenness estimates^18^. No difference in alpha diversity estimates was observed among the faecal samples, corresponding KUHIMMs and corresponding KUHIMMs with DBHB administration (Mann-Whitney U test; *p* > 0.05). The average bacterial community compositions at the genus level were determined in the original faeces, corresponding KUHIMMs and corresponding KUHIMMs with DBHB administration (Fig. 2). Interestingly, comparisons between KUHIMMs with and without DBHB revealed that DBHB administration increased the relative proportion of bacteria related to the genus *Coprococcus* (Wilcoxon signed-rank test; *p* = 0.031; see Supplementary Fig. S1). In contrast, DBHB administration did not increase the relative proportion of bacteria related to the genus *Coprococcus* in the three non-utiliser KUHIMMs (Wilcoxon signed-rank test; *p* = 0.750; Fig. 2). On the other hand, DBHB utilisation was not found in pure cultures of *Coprococcus comes* or *Coprococcus eutactus*.

**Table 1:** Summary of 16S rRNA gene sequencing data and α-diversity values (Chao1, Shannon index and Simpson index) of the D-β-hydroxybutyrate (DBHB) utilisers and non-utilisers.

	Faeces	KUHIMM	+DBHB
		−DBHB	
**Utiliser (n = 7)**			
Read counts	158,058 ± 35,293	158,104 ± 44,838	154,368 ± 44,768
Observed OTUs	1,223 ± 364	1,127 ± 238	1,038 ± 234
Chao1	2,905 ± 711	2,853 ± 717	2,736 ± 643
Shannon index	5.93 ± 0.76	5.53 ± 0.55	5.36 ± 0.56
Simpson index	0.95 ± 0.03	0.95 ± 0.02	0.94 ± 0.02
**Non-utiliser (n = 3)**			
Read counts	203,060 ± 18,474	216,118 ± 35,167	170,497 ± 36,869
Observed OTUs	1,391 ± 400	1,117 ± 95	757 ± 453
Chao1	3,003 ± 459	2,389 ± 461	1,850 ± 1,194
Shannon index	5.80 ± 0.35	4.96 ± 0.43	3.84 ± 1.90
Simpson index	0.95 ± 0.01	0.91 ± 0.03	0.74 ± 0.29

Human faecal samples, the corresponding KUHIMMs and the corresponding KUHIMM with sodium D-β-hydroxybutyrate (DBHB) were sampled at 30 h of fermentation. The values signify the mean ± standard deviation.

**Figure 3:**
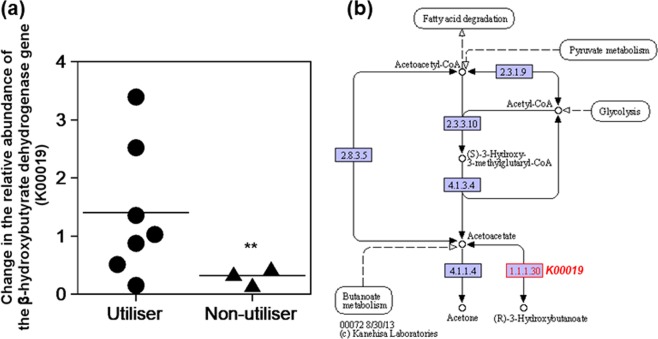
Changes in the relative abundance of the β-hydroxybutyrate dehydrogenase gene (*K00019*). (**a**) The functional abundance was compared between the corresponding *in vitro* microbiota models (KUHIMMs) with and without D-β-hydroxybutyrate (DBHB) addition after 30 h of fermentation with the original faecal inoculum (+DBHB / -DBHB). DBHB utilisers (n = 7) and non-utilisers (n = 3) are shown. **p < 0.01; Dunnett test. The result is based on estimation from the PICRUSt analysis. (**b**) Pathway map of synthesis and degradation of ketone bodies. Map (ko00072) was cited from https://www.kegg.jp/kegg/kegg1.html with permission.

The factor(s) underlying the difference in DBHB availability in the KUHIMMs was explored. To understand the potential implications of the different KUHIMMs, we performed PICRUSt analysis, a computational approach that predicts the functional composition of the bacterial metagenome using 16S rRNA data^19^. The first step in DBHB utilisation is its conversion to acetoacetate by β-hydroxybutyrate dehydrogenase^20^. Thus, the relative abundance of the β-hydroxybutyrate dehydrogenase gene (*K00019*) was compared among the faecal samples, corresponding KUHIMMs and corresponding KUHIMMs with DBHB administration (including both utiliser and non-utiliser KUHIMMs; see Supplementary Fig. S2). The relative abundance of the β-hydroxybutyrate dehydrogenase gene was not significantly changed by DBHB addition in the DBHB utilisers (Dunnett test; *p* = 0.370); in contrast, the relative abundance of the β-hydroxybutyrate dehydrogenase gene expression was significantly decreased in the DBHB non-utilisers (Dunnett test; *p* = 0.001; Fig. 3).

### Increased butyrogenesis following D-β-hydroxybutyrate addition in utilisers

**Figure 4:**
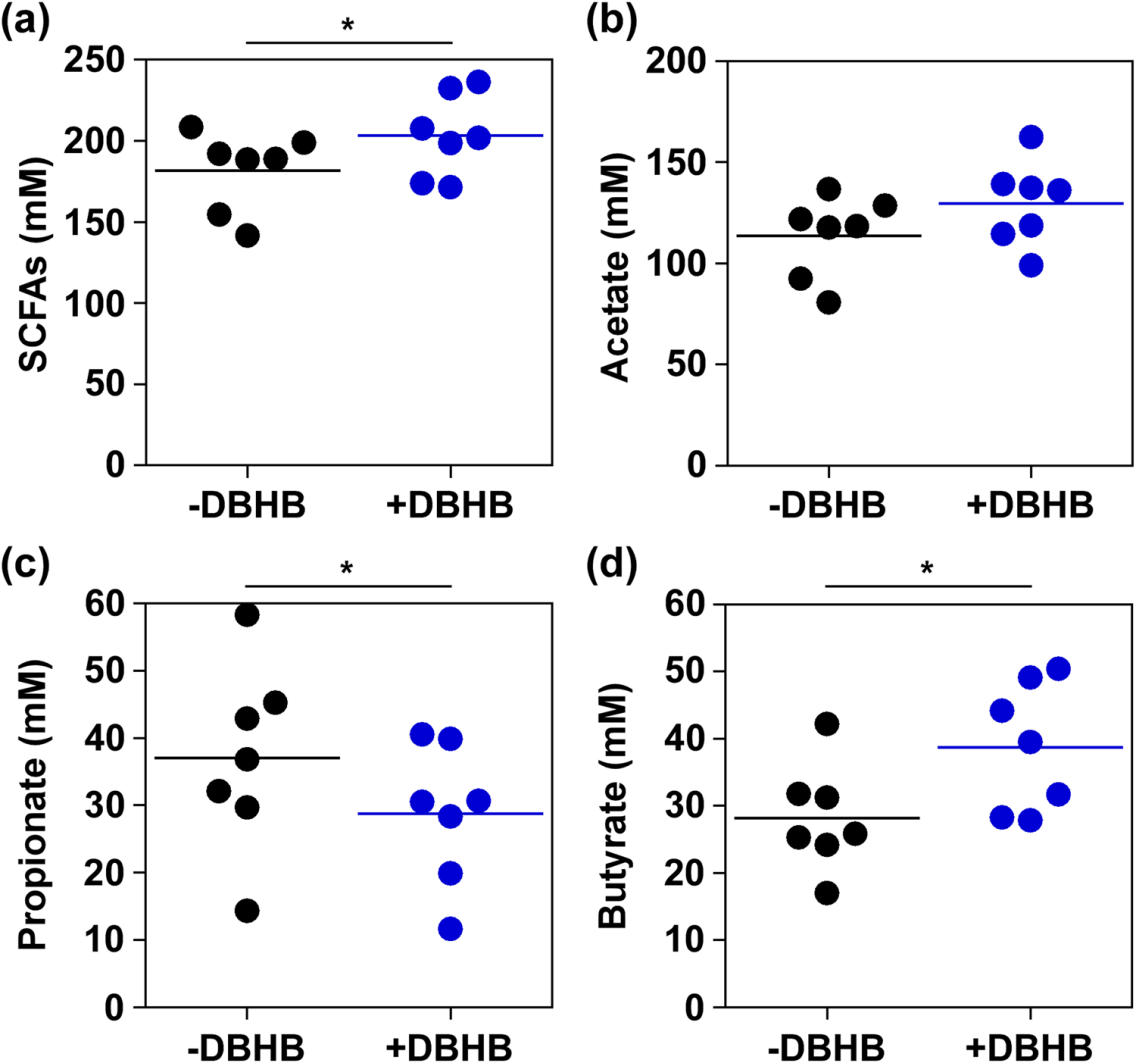
Volatile fatty acid production in D-β-hydroxybutyrate (DBHB) utilisers (n = 7) in *in vitro* microbiota models (KUHIMMs) after 30 h of fermentation with the original faecal inoculum. SCFAs (sum of lactate, succinate, acetate, propionate and butyrate). *Significant differences between those with DBHB (+DBHB) and without DBHB (-DBHB); *p < 0.05.

To analyse the function of the colonic microbiota, we compared the production of short chain fatty acids (SCFAs) by KUHIMMs with and without sodium DBHB after 30 h of fermentation with the original faecal inoculum. Interestingly, butyrate production was increased by DBHB addition (Wilcoxon signed-rank test; *p* = 0.031) in the utilisers (n = 7). In addition, an increase in SCFA production (sum of lactate, succinate, acetate, propionate, and butyrate) was observed (Wilcoxon signed-rank test; *p* = 0.016; Fig. 4) and propionate production was decreased (Wilcoxon signed-rank test; *p* = 0.031). In contrast, DBHB addition did not significantly affect SCFA production in the non-utilisers (n = 5; Wilcoxon signed-rank test; *p* = 0.063) (see Supplementary Fig. S3).

### KUHIMM metabolomics

**Figure 5:**
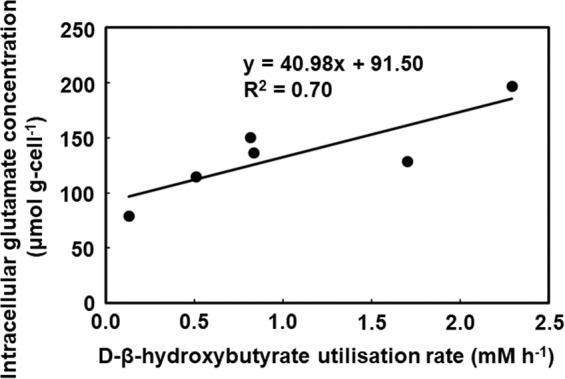
Relationship between the D-β-hydroxybutyrate utilisation rate (mM h^−1^) and intracellular glutamate concentration (µmol g-cell^−1^) after 21 h of fermentation with the original faecal inoculum (n = 6).

To investigate the physiological state of the microorganisms^21^, the metabolomics of six KUHIMMs were examined after 21 h of fermentation with the original faecal inoculum. The DBHB utilisation rate was simultaneously calculated and compared with the concentrations of intracellular metabolites obtained from the KUHIMMs. Interestingly, the intracellular glutamate concentration showed a positive correlation with the DBHB utilisation rate (R^2^ = 0.70; Fig. 5).

## Discussion

Administration of exogenous ketones and ketone bodies can be used in the treatment of several neurological diseases^22^. A recent study has shown that, in the field of cancer immunotherapy, the gut microbiome influences the effect of immune checkpoint inhibitors on patients^23^. Desirably, the administration of DBHB should have a positive effect on the human gut microbiota. *In vitro* fermentation of human faecal inoculum was used to construct the colonic microbiota model, KUHIMM, to maintain most of the OTUs in faeces. However, differences from faecal inoculum such as an increase in *Enterobacteriaceae* occurred, as described previously^24^. Our results using the KUHIMM suggested that more than half (seven of twelve) of the microbiota in human subjects could utilise DBHB. In DBHB utilisers, the increase in the relative proportion of bacteria related to the genus *Coprococcus* following DBHB administration was correlated with an increase in butyrogenesis. This result is logical, as the major butyrate-producing bacteria isolated from the human colon have been reported to include bacteria related to the genus *Coprococcus*^25^. Butyrate is produced from DBHB via acetoacetyl-CoA, β-hydroxybutyryl-CoA and crotonyl-CoA^26^, and is thought to benefit health including protection against colorectal cancer^27^. In addition, butyrate treatment has been shown to have an effect similar to DBHB, lowering glucose and insulin levels, improving glucose tolerance and preventing weight gain via histone deacetylase inhibition^20,28^. However, increased butyrogenesis was not observed in the five non-utilisers of DBHB, probably because there was no increase in the relative proportion of bacteria related to *Coprococcus* and Unclassified *Lachnospiraceae*, which reportedly produce butyrate^29^. Thus, a combination of ketone supplementation with SCFAs, such as butyrate, may be a more appropriate treatment for complementing the gut microbiota in non-utilisers.

In this work, the functional composition of the microbial community was predicted from 16S data using PICRUSt following a previously reported method, although gene expression was not studied^30^. The following two factors determined the efficiency of DBHB utilisation by the human colonic microbiota: first, the relative abundance of the β-hydroxybutyrate dehydrogenase gene remained unchanged following DBHB administration in the utilisers, compared with non-utilisers; second, the intracellular glutamate concentration was higher in the colonic microbiota of utilisers than the non-utilisers. It is logical that expression of β-hydroxybutyrate dehydrogenase, which is involved in the first step of DBHB utilisation^20^, is maintained in the colonic microbiota of the utilisers. Interestingly, the glutamate content in the colonic microbiota was well correlated with the DBHB utilisation rate. To the best of our knowledge, this is the first time that such a correlation was observed. In the colonic microbiota of utilisers, DBHB enters the tricarboxylic acid (TCA) cycle where it is metabolised to glutamate via α-ketoglutarate. Glutamate is a key metabolite linking nitrogen and carbon metabolism, i.e., anabolism and catabolism, in all living organisms^31^, and serves as the general amino group donor for amino acid and nucleotide biosynthesis^32^. In addition, our previous study suggested that the intracellular glutamate concentration is an indicator of bacterial consortium growth^33^. The human colonic microbiota might have higher growth activity in DBHB utilisers than in non-utilisers.

Our results indicate that the human colonic microbiota is divided into the DBHB utilisers and non-utilisers. The colonic microbiota in DBHB utilisers is capable of enhancing the generation of butyrate, which would have beneficial effects on human health, and growth. Future studies should investigate whether utilisation or non-utilisation of DBHB is reproducible *in vivo* and how these differences in the colonic microbiota affect DBHB treatment in patients with neurological diseases.

## Materials and methods

### Preparation of D-β-hydroxybutyrate sodium salt

DBHB was produced and purified from strain *Halomonas* sp. KM-1, as previously described^34^, and neutralised with NaOH.

### Preparation of faecal inoculums

Fresh faecal samples were obtained from 12 healthy Japanese volunteers, in their early twenties to late fifties (see Supplementary Table S1 online). None of these individuals had a history of antibiotic treatment in the six months prior to the study. All subjects provided written informed consent prior to specimen collection. Immediately following collection, each faecal sample was stored in an anaerobic culture swab (212550 BD BBL, Culture Swab; Becton, Dickinson and Company, Franklin Lakes, NJ, USA) and used within 24 h. The study was performed in accordance with the guidelines of Kobe University Hospital and was approved by the institutional ethics review board of Kobe University. All methods used in this study were in accordance with the Declaration of Helsinki.

### KUHIMM construction

The KUHIMMs were constructed, with or without the addition of sodium DBHB, using a multi-channel fermenter (Bio Jr. 8; ABLE, Tokyo, Japan), as previously described^17^. Each fermenter contained autoclaved Gifu anaerobic medium (GAM [Code 05422]; Nissui Pharmaceutical Co, Tokyo, Japan), with an initial pH adjusted to 6.5. The medium was maintained at 37 °C with constant stirring at 300 rpm and anaerobiosis was maintained by continuous flow (15 mL min^−1^) of a filter-sterilised N_2_:CO_2_ (80:20) gas blend prior to cultivation and during fermentation. To evaluate the effect of DBHB, sodium DBHB was added into one of the 100-mL fermenters at a final concentration of 0.5% (w/v) [corresponding to 0.41% (w/v) of DBHB] prior to cultivation. A corresponding control fermenter without sodium DBHB was also prepared. Each faecal sample was suspended in 0.1 M phosphate buffer (pH 6.5, consisting of a 7:3 mixture of 0.1 M NaH_2_PO_4_ and 0.1 M Na_2_HPO_4_) supplemented with 1% L-ascorbic acid (Wako Pure Chemical Industries, Osaka, Japan). Each set of two fermenters was inoculated with 100 μL of the abovementioned faecal suspension. Culture supernatant aliquots (1 mL) were sampled from the fermenters at 30 h after initiation of fermentation. Faecal and fermentation broth samples were stored at −20 °C until use.

### Microbial DNA extraction

Microbial genomic DNA was extracted from the suspended faecal samples and cultured KUHIMMs at 30 h, as previously described^35^. Purified DNA was eluted in TE buffer (10 mM Tris-HCl, 1.0 mM ethylenediaminetetraacetic acid) and stored at −20 °C until use.

### Illumina library generation

Bacterial 16 S rRNA genes (V3-V4 region) were amplified using genomic DNA as the template and the primers S-D-Bact-0341-b-S-17 (5′-CCTACGGGNGGCWGCAG-3′) and S-D-Bact-0785-a-A-21 (5′-GACTACHVGGGTATCTAATCC-3′)^36^, as previously described^17^. Illumina adapter overhang nucleotide sequences were added to the gene-specific sequences. Polymerase chain reaction and amplicon pool preparation were performed according to the manufacturer’s instructions. Amplicons were purified using AMPure XP DNA purification beads (Beckman Coulter, Brea, CA, USA), and eluted in 25 µL of 10 mM Tris (pH 8.5). Purified amplicons were quantified using an Agilent Bioanalyzer 2100 with DNA 1000 chips (Agilent Technology, Santa Clara, CA, USA) and a Qubit 2.0 instrument (Thermo Fisher Inc., Waltham, MA, USA), and pooled at equimolar concentrations (5 nM). The 16S rRNA genes and an internal control (PhiX control v3; Illumina) were subjected to paired-end sequencing using a MiSeq instrument (Illumina) and the MiSeq Reagent Kit, v3 (600 cycles; Illumina). The PhiX sequences were removed, and paired-end reads with Q scores ≥ 20 were joined using the MacQIIME software package, version 1.9.1^37^. The UCLUST algorithm^38^ was used to cluster the filtered sequences into OTUs based on a ≥97% similarity threshold. Chimeric sequences were identified and removed from the library using ChimeraSlayer^39^. Representative sequences from each OTU were taxonomically classified via the GreenGenes taxonomy database using the Ribosomal Database Project Classifier^40^. Functional analysis of gut microbiota based on 16S rRNA gene sequences and the KEGG Orthology database (Kyoto Encyclopedia of Genes and Genomes)^41^ was performed using PICRUSt 1.1.1^19^. Pathway map of synthesis and degradation of ketone bodies (ko00072) was cited from https://www.kegg.jp/kegg/kegg1.html with permission.

### Measurement of SCFA and sodium D-β-hydroxybutyrate concentrations

The concentrations of SCFAs, such as acetate, propionate, butyrate, lactate and succinate, and sodium DBHB, were measured using a high-performance liquid chromatography (HPLC) instrument (Shimadzu Corporation, Kyoto, Japan) equipped with an Aminex HPX-87H column (Bio-Rad Laboratories, Inc., Hercules, CA, USA) and a RID-10A refractive index detector (Shimadzu Corporation) as described previously^35^. The HPLC instrument was operated at 65 °C using 5 mM H_2_SO_4_ as the mobile phase with a flow rate of 0.6 mL min^−1^.

### Culture of *Coprococcus* strains

*Coprococcus comes* JCM 31264 and *Coprococcus eutactus* JCM 31265 were obtained from the Japan Collection of Microorganisms (JCM). *Coprococcus* spp. were cultured in modified reinforced clostridial medium (RCM) at 37 °C under anaerobic conditions (10% H_2_, 10% CO_2_, and 80% N_2_) for 2 days. Modified RCM was prepared by dissolving 10 g of Bacto Peptone, 13 g of yeast extract, 5 g of glucose, 1 g of soluble starch, 5 g of sodium chloride, 3 g of sodium acetate, and 0.5 g of _L_-cysteine in 1 L of deionized distilled water. For comparison, *Coprococcus* spp. were cultured similarly by additionally dissolving 5 g of sodium DBHB in 1 L of deionized distilled water.

### Intracellular metabolite extraction, quenching and mass spectrometry

Cells were collected from the KUHIMM culture supernatant with sodium DBHB at 21 h after the initiation of fermentation. KUHIMMs in which utilisation of DBHB was not observed at 21 h and in which the DBHB utilisation rate could not be calculated, were excluded from the metabolomics analysis. Each of the fermenters was inoculated with one of six faecal suspensions. The cell weight of each sample was adjusted to the same level based on the optical density at 600 nm (OD_600_) before filtering each sample through a 4-polytetrafluoroethylene membrane filter (Omnipore, 0.45 µM, 47-mm diameter; Millipore, Billerica, MA, USA) as described previously^33^. The dry cell weight of each sample was estimated by multiplying the determined cell weight of *Escherichia coli* at OD_600_ using the equation shown in Eq. (1).

1 $${\rm{Dry}}\,{\rm{cell}}\,{\rm{weight}}\,({\rm{mg}})\,=\,0.0582\times {{\rm{OD}}}_{600}\times {\rm{cell}}\,{\rm{suspension}}\,({\rm{\mu }}{\rm{L}})$$

Immediately after filtration, the cells were washed with cold phosphate-buffered saline (PBS: 137 mM NaCl, 8.10 mM Na_2_HPO_4_, 2.68 mM KCl and 1.47 mM KH_2_PO_4_). Membrane filters with the washed cells were transferred into 50-mL centrifuge tubes and then frozen in liquid nitrogen. Metabolites were extracted from the cells using a modified cold chloroform-methanol method^42^. Finally, the water phase of the extract (700 µL) was dried under vacuum and stored at −80 °C until further analysis^43^.

The dried extract samples were thawed on ice, derivatised at 30 °C for 90 min with 100 µL of 20 mg mL^−1^ methoxyamine hydrochloride in pyridine, after which 50 µL of N-methyl-N-(trimethylsilyl) trifluoroacetamide (GL Sciences, Tokyo, Japan)^44^ were added, followed by incubation at 37 °C for 30 min. Derivatised samples (1 µL) were subjected to gas chromatography-quadrupole-mass spectrometry (GC-Q-MS) using a GCMSQP-2010 system (Shimadzu) to detect metabolites.

### Bioinformatics and statistical analyses

The α-diversity values (Chao1, Shannon index and Simpson index) were calculated using the MacQIIME software package^37^. Data were analysed using the Mann-Whitney U test, Wilcoxon signed-rank test (two-sided) and Dunnett test using the JMP 12 software (SAS Institute Inc., Cary, NC, USA). *p* < 0.05 was considered statistically significant.

## Supplementary information

Supplementary information.

## Data Availability

All raw sequence data generated in this study have been deposited on the MG-RAST server^45^ (http://metagenomics.anl.gov) in a file named “Model Culture System of Human Colonic Microbiota_DBHB” under accession numbers mgm4869957.3 – mgm4869986.3.

## References

[1] Grabacka M, Pierzchalska M, Dean M, Reiss K (2016). Regulation of ketone body metabolism and the role of PPARα. Int. J. Mol. Sci..

[2] Puchalska P, Crawford PA (2017). Multi-dimensional roles of ketone bodies in fuel metabolism, signaling, and therapeutics. Cell Metab..

[3] Martin K, Jackson CF, Levy RG, Cooper PN (2016). Ketogenic diet and other dietary treatments for epilepsy. Cochrane Database Syst. Rev..

[4] McDonald TJW, Cervenka MC (2018). The expanding role of ketogenic diets in adult neurological disorders. Brain Sci.

[5] Ye F, Li XJ, Jiang WL, Sun HB, Liu J (2015). Efficacy of and patient compliance with a ketogenic diet in adults with intractable epilepsy: a meta-analysis. J. Clin. Neurol..

[6] Kovács Z (2019). Therapeutic potential of exogenous ketone supplement induced ketosis in the treatment of psychiatric disorders: review of current literature. Front Psychiatry.

[7] Gormsen LC (2017). Ketone body infusion with 3-hydroxybutyrate reduces myocardial glucose uptake and increases blood flow in humans: a positron emission tomography study. J. Am. Heart Assoc.

[8] Fischer T (2018). Effect of sodium and calcium DL-β-hydroxybutyrate salt in healthy adults. J. Nutr. Metab.

[9] Si J (2017). Anticonvulsant effect of exogenous β-hydroxybutyrate on kainic acid-induced epilepsy. Exp. Ther. Med.

[10] Nicholson JK (2012). Host-gut microbiota metabolic interactions. Science.

[11] Xie G (2017). Ketogenic diet poses a significant effect on imbalanced gut microbiota in infants with refractory epilepsy. World J. Gastroenterol..

[12] Newell C (2016). Ketogenic diet modifies the gut microbiota in a murine model of autism spectrum disorder. Mol. Autism.

[13] Olson CA (2018). The gut microbiota mediates the anti-seizure effects of the ketogenic diet. Cell..

[14] Zhang Y (2018). Altered gut microbiome composition in children with refractory epilepsy after ketogenic diet. Epilepsy Res..

[15] Ma D (2018). Ketogenic diet enhances neurovascular function with altered gut microbiome in young healthy mice. Sci. Rep.

[16] Duan Y, Zhang Y, Dong H, Wang Y, Zhang J (2017). Effect of dietary poly-β-hydroxybutyrate (PHB) on microbiota composition and the mTOR signaling pathway in the intestines of *Litopenaeus vannamei*. J. Microbiol..

[17] Sasaki D (2018). Low amounts of dietary fibre increase *in vitro* production of short-chain fatty acids without changing human colonic microbiota structure. Sci. Rep.

[18] Sinclair L, Osman OA, Bertilsson S, Eiler A (2015). Microbial community composition and diversity via 16S rRNA gene amplicons: evaluating the illumine platform. PLoS One.

[19] Langille MG (2013). Predictive functional profiling of microbial communities using 16S rRNA marker gene sequences. Nat. Biotechnol..

[20] Newman JC, Verdin E (2014). β-hydroxybutyrate: much more than a metabolite. Diabetes Res. Clin. Pract..

[21] Tang J (2011). Microbial metabolomics. Curr. Genomics..

[22] Yang H, Shan W, Zhu F, Wu J, Wang Q (2019). Ketone bodies in neurological diseases: focus on neuroprotection and underlying mechanisms. Front Neurol.

[23] Routy B (2018). Gut microbiome influences efficacy of PD-1-based immunotherapy against epithelial tumors. Science.

[24] Sasaki K (2019). Construction of a model culture system of human colonic microbiota to detect decreased *Lachnospiraceae* abundance and butyrogenesis in the feces of ulcerative colitis patients. Biotechnol. J.

[25] Louis P, Flint HJ (2009). Diversity, metabolism and microbial ecology of butyrate-producing bacteria from the human large intestine. FEMS Microbiol. Lett.

[26] Louis P, Flint HJ (2017). Formation of propionate and butyrate by the human colonic microbiota. Environ. Microbiol..

[27] Thanikachalam K, Khan G (2019). Colorectal cancer and nutrition. Nutrients.

[28] Gao Z (2009). Butyrate improves insulin sensitivity and increases energy expenditure in mice. Diabetes.

[29] Vital M, Karch A, Pieper DH (2017). Colonic butyrate-producing communities in humans: an overview using omics data. mSystems..

[30] Frankenfeld CL, Sikaroodi M, Lamb E, Shoemaker S, Gillevet PM (2015). High-intensity sweetner consumption and gut microbiome content and predicted gene function in a cross-sectional study of adults in the united states. Ann. Epidemiol..

[31] Feehily C, Karatzas KA (2013). Role of glutamate metabolism in bacterial responses toward acid and other stresses. J. Appl. Microbiol..

[32] Communichau FM, Forchhammer K, Stülke J (2006). Regulatory links between carbon and nitrogen metabolism. Curr. Opin. Microbiol..

[33] Sasaki D, Sasaki K, Tsuge Y, Kondo A (2019). Less biomass and intracellular glutamate in anodic biofilms lead to efficient electricity generation by microbial fuel cells. Biotechnol. Biofuels.

[34] Kawata Y, Ando H, Matsushita I, Tsubota J (2014). Efficient secretion of (R)-3-hydroxybutyric acid from *Halomonas* sp. KM-1 by nitrate fed-batch cultivation with glucose under microaerobic conditions. Bioresour. Technol.

[35] Takagi R (2016). A single-batch fermentation system to simulate human colonic microbiota for high-throughput evaluation of prebiotics. Plos One.

[36] Klindworth A (2013). Evaluation of general 16S ribosomal RNA gene PCR primers for classical and next-generation sequencing-based diversity studies. Nucleic Acids Res..

[37] Caporaso JG (2010). QIIME allows analysis of high-throughput community sequencing data. Nat. Methods.

[38] Edgar RC (2010). Search and clustering orders of magnitude faster than BLAST. Bioinformatics.

[39] Haas BJ (2011). Chimeric 16S rRNA sequence formation and detection in Sanger and 454-pyrosequenced PCR amplicons. Genome Res..

[40] Wang Q, Garrity GM, Tiedje JM, Cole JR (2007). Naive Bayesian classifier for rapid assignment of rRNA sequences into the new bacterial taxonomy. Appl. Environ. Microbiol..

[41] Kanehisa M, Furumichi M, Tanabe M, Sato Y, Morishima K (2017). KEGG: new perspectives on genomes, pathways, diseases and drugs. Nucleic Acids Res.

[42] Putri SP (2013). Current metabolomics: practical applications. J. Biosci. Bioeng..

[43] Bennett BD, Yuan J, Kimball EH, Rabinowitz JD (2008). Absolute quantitation of intracellular metabolite concentrations by an isotope ratio-based approach. Nat. Protoc..

[44] Lisec J, Schauer N, Kopka J, Willmitzer L, Fernie AR (2006). Gas chromatography mass spectrometry-based metabolite profiling in plants. Nat. Protoc..

[45] Meyer F (2008). The metagenomics RAST server – a public resource for the automatic phylogenetic and functional analysis of metagenomes. BMC Bioinformatics..

