# Supplementary material for: In vitro human colonic microbiota utilises D-β-hydroxybutyrate to increase butyrogenesis
Source: Sci Rep. 2020 May 22;10:8516. doi: 10.1038/s41598-020-65561-5 (PMC7244492; doi:10.1038/s41598-020-65561-5)
Supplement: Supplementary file 1 — Supplementary information. [file 41598_2020_65561_MOESM1_ESM.docx]

**Title**

*In vitro* human colonic microbiota utilises D-β-hydroxybutyrate to increase butyrogenesis

**Authors**

Kengo Sasaki^1^, Daisuke Sasaki^1^, Asuka Hannya^2^, Jun Tsubota^2^, and Akihiko Kondo^1,3^*

^1^Graduate School of Science, Technology and Innovation, Kobe University, 1-1 Rokkodai-cho, Nada-ku, Kobe, Hyogo 657-8501, Japan

^2^Energy Technology Laboratories, OSAKA GAS CO., LTD., 6-19-9 Torishima, Konohana-ku, Osaka 554-0051, Japan

^3^RIKEN Center for Sustainable Resource Science, 1-7-22 Suehiro-cho, Tsurumi-ku, Yokohama, Kanagawa 230-0045, Japan

*Correspondence and requests for materials should be addressed to K.S. (email: sikengo@people.kobe-u.ac.jp)


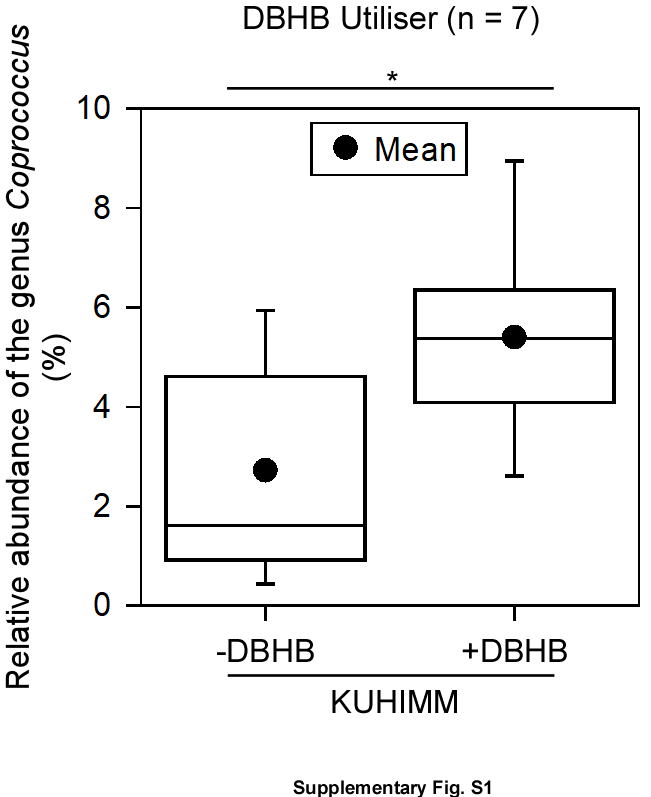


**Supplementary Fig. S1.** Relative abundance of bacteria related to the genus *Coprococcus* in seven *in vitro* human colonic microbiota models (KUHIMMs) classified as of D-β-hydroxybutyrate (DBHB) utilisers. The black circles indicate the mean value.


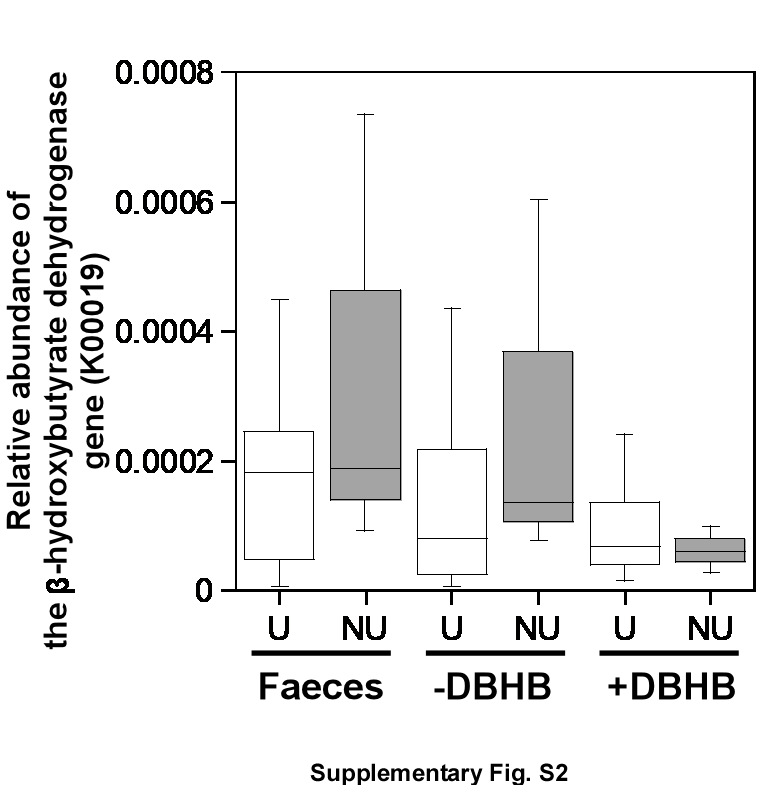


**Supplementary Fig. S2.** Relative abundance of the β-hydroxybutyrate dehydrogenase gene (*K00019*) in the original faecal samples (Faeces), the corresponding *in vitro* microbiota models, KUHIMMs (-DBHB), and the corresponding KUHIMMs with sodium D-β-hydroxybutyrate (+DBHB) after 30 h of fermentation. DBHB utilisers (U, n = 7) and non-utilisers (NU, n = 3) are shown.


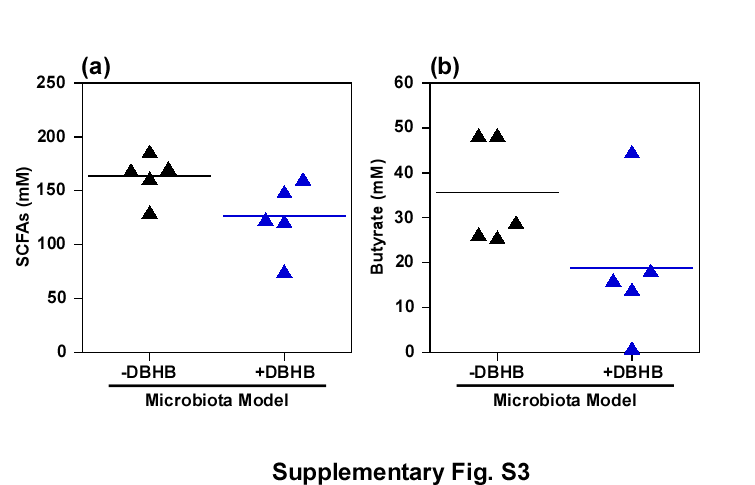


**Supplementary Fig. S3.** Volatile fatty acid production by D-β-hydroxybutyrate non-utilisers (n = 5) in *in vitro* microbiota models (KUHIMMs) after 30 h of fermentation with the original faecal inoculum. SCFAs (sum of lactate, succinate, acetate, propionate and butyrate).

**Supplementary Table S1.** Characteristics of study volunteers comprising utilisers and non-utilisers of D-β-hydroxybutyrate (DBHB).

|  | Utiliser (n = 7) | Non-utiliser (n = 5) |
| --- | --- | --- |
| Age in years [median (range)] | 40 (34–45) | 38 (21–57) |
| Sex (F/M) | (3/4) | (3/2) |
